# Insect in the Ear—Response and Treatment of an Uncommon Prehospital Emergency: A Case Report

**DOI:** 10.5811/cpcem.48649

**Published:** 2025-11-26

**Authors:** Colin Bashline, Matthew Jester, Christopher Morris

**Affiliations:** * Ross/West View EMSA, Pittsburgh, Pennsylvania; † Allegheny General Hospital, Department of Emergency Medicine, Pittsburgh, Pennsylvania

**Keywords:** foreign body, external auditory canal, prehospital care, emergency medical services, case report

## Abstract

**Introduction:**

Foreign bodies in the external auditory canal are an uncommon presentation in emergency settings. Among adults, insects represent a frequent organic foreign body, often causing symptoms such as otalgia, tinnitus, vertigo, and anxiety. Prehospital management of such cases is rarely addressed in the medical literature, with minimal guidance available for emergency medical services (EMS) personnel. In this report we discuss their role in stabilizing patients and reducing discomfort through appropriate interventions.

**Case Report:**

A 40-year-old male called EMS after a live insect entered his left ear, causing severe otalgia and distress. Prehospital medical personnel clinically confirmed the presence of the insect and assessed for signs of tympanic membrane perforation. A medical command physician authorized the use of 2% lidocaine to euthanize the insect, which alleviated movement-related discomfort within 20 seconds. Despite initial symptom relief, the patient experienced persistent fullness in the ear and was transported to a tertiary-care hospital. In the emergency department multiple removal attempts were made, with successful extraction using thin dressing forceps. No tympanic membrane perforation was noted, although minor trauma to the external auditory canal was present. The patient was discharged with ciprofloxacin-dexamethasone otic drops and return precautions.

**Conclusion:**

Prehospital use of lidocaine for a live insect in the auditory canal may provide significant symptom relief while reducing the risk of further auditory canal trauma. This case underscores the importance of command-based support for EMS personnel to provide safe, evidence-based approaches for managing intra-aural insects in the field.

## INTRODUCTION

Foreign bodies of the external auditory canal are an infrequent but uniquely uncomfortable presentation to the emergency department (ED). Patients presenting with tinnitus, vertigo, otalgia, otorrhea, decreased hearing, hyperacusis and, in rare cases, persistent cough or hiccups should be evaluated for foreign bodies.^1^ Literature detailing the treatment of such patients is sparse and largely anecdotal. Prehospital guidance is limited, consisting of a handful of wilderness medicine guidelines.^2^,^3^

## CASE REPORT

We present a case of a 40-year-old male who called emergency medical services (EMS) for a live insect that had flown into his left ear while he was in his garage. The patient complained of otalgia from the insect trying to move or fly. His spouse had unsuccessfully attempted to visualize the insect in the auditory canal prior to EMS arrival. Responders’ visual inspection showed no outward abnormalities of the ear and no drainage or bleeding from the auditory canal, but the patient was restless and anxious. Persisting symptoms suggested that the insect was still in the auditory canal, prompting EMS to evaluate vital signs and contact a medical command physician for treatment orders.

After discussion with medical command to rule out signs suggesting a perforated tympanic membrane, such as hearing loss or bloody otorrhea, the EMS responders performed a lidocaine lavage. The patient’s head was rotated such that his left ear pointed up, and 2% lidocaine solution was instilled until the external auditory canal was filled. The patient was instructed to inform the EMS responders when the sensation of movement ceased. After approximately 20 seconds the movement ceased, and the patient’s head was then returned to anatomical position. The lidocaine drained by gravity and was absorbed with a paper towel. The external ear was inspected by flashlight, which yielded what appeared to be a single, detached antenna. Due to lack of visualization of the insect, the EMS responders made no attempt to remove it. While the patient’s distress and pain had subsided, a sensation of fullness remained in the left ear. He was transported to a nearby tertiary-care hospital for removal of the dead insect.

Additional history from the patient revealed that he initially attempted to remove the insect with his finger. This action caused the insect to be further wedged inside his external auditory canal. On arrival to the hospital, the patient was still very uncomfortable. An initial attempt to remove the insect with a cerumen loop was unsuccessful. A second, successful attempt entailed removing the insect (Image) with thin dressing forceps. There was no associated perforated tympanic membrane; however, the patient had minor trauma to the auditory canal. He was discharged with ciprofloxacin-dexamethasone 0.3–0.1% otic suspension drops and strict return precautions. Patient comfort during the extraction process had been improved as a result of the topical analgesia used by the paramedics.

*CPC-EM Capsule*

What do we already know about this clinical entity?

*Using lidocaine to kill a live insect in the ear prior to removal normally occurs in a hospital setting.*

What makes this presentation of disease reportable?

*We document prehospital treatment by emergency medical services responders who used lidocaine to kill an insect in a patient’s auditory canal before transporting him to the hospital.*

What is the major learning point?

*With a low clinical suspicion for tympanic membrane rupture, prehospital personnel should consult with medical command physicians and consider use of lidocaine.*

How might this improve emergency medicine practice?

*An animate foreign body in the ear is extremely uncomfortable. Killing the insect in the field minimizes suffering while the patient is transported to definitive medical care.*

## DISCUSSION

Foreign bodies in the auditory canal are more common in pediatric patients and occur more frequently in males.^1^,^4^ While most of the foreign bodies seen in children are inorganic and iatrogenic, such as beads, toys and batteries, some of these objects are insects. As patient age increases the proportion of organic foreign bodies increases, with insects one of the most common culprits in adults.^1^ Insect foreign bodies tend to be isolated and accidental.

Insects pose a unique concern compared to other foreign bodies due to their ability to move. Insects tend to have difficulty walking backwards, which causes them to become trapped in the patient’s ear canal.^5^,^7^ After becoming stuck, the insects tend to move erratically, which can cause distressing sound and sensations, as well as pain. Multiple case reports have shown the significant and potentially fatal sequelae that insects in the ear can pose. Damage from insects in the middle and inner ear can lead to mastoiditis or facial nerve palsies either by direct trauma or localized swelling.^6^,^8^ A 2020 case report from India described a patient who suffered from skull base osteomyelitis and mucormycosis that led to thrombi, neurological deficit, and death after two days of intensive treatment.^7^,^9^ While extreme, these complications illustrate the potential for animate foreign bodies to produce life-threatening sequelae.

A key point to emphasize is the need for prompt referral to professional care. While emergency physicians are considered appropriate for initial removal attempts, there is strong preference for referral to otolaryngologists if initial attempts fail, or if timely initial referral can be made.^4^,^6^,^8^ While most patients do seek professional care, clinicians should be wary of the inclination to attempt home remedy; attempts at removal of insects have been reported to include cotton swabs, whole cloves of garlic, and hot oil poured into the auditory canal. These home remedies can result in worsening impaction, infection, burns, and trauma to the external auditory canal and tympanic membrane.^4^

While the need to remove the insect is obvious, the first step of treatment should focus on killing the insect to prevent further damage and reduce pain and distress.^1^,^3^,^8^,^10^ Multiple options exist for dispatching the insect, with the primary choices being mineral oil and lidocaine. A unique case report from Pittsburgh reported a patient with cockroaches in both ears.^9^,^11^ Clinicians used this opportunity to compare the efficacy of mineral oil vs lidocaine solution; the mineral oil swiftly killed the insect but made physical removal difficult.^9^,^11^ The lidocaine solution, by contrast, caused the roach to exit the ear canal “at a convulsive rate of speed.”^9^,^11^

The rates of efficacy of these two solutions have been evaluated by researchers under more controlled circumstances. Leffler et al evaluated 40 American cockroaches that had been administered mineral oil and varying concentrations of lidocaine; they concluded that mineral oil was significantly faster at killing the roaches, with lidocaine taking > 10 seconds longer.^10^,^12^ Differences in concentration did not seem to affect the speed at which lidocaine killed the roaches.^10^,^12^ Use of water was ruled out, as it took > two minutes to act.^10^,^12^ A study from Antonelli et al evaluated multiple solutions, finding ethanol and isopropyl alcohol to be the most effective agents for killing insects.^11^ However, mineral oil was still found to be more effective than lidocaine.^11^,^13^

Based on these studies and multiple anecdotal cases, mineral oil and 1–2% non-viscous lidocaine are the two most effective and readily available agents for killing an insect in the external auditory canal.^2^,^8^,^10^,^11^,^13^ Lidocaine is considered to have added benefit in its anesthetic properties. We recommend that caution and close discussion with medical command physicians be used to clinically evaluate risk of perforation of the tympanic membrane. Use of lidocaine with a perforated tympanic membrane can cause interactions with the membranous labyrinth, leading to severe vertigo and subsequent nausea and vomiting.^2^,^3^,^11^,^13^ While both mineral oil and lidocaine would be effective in killing the insect and bringing immediate relief to the patients, mineral oil is not routinely stocked in ambulances; more importantly, lidocaine can be ototoxic if the tympanic membrane is perforated.^12^,^14^ Given this potential for toxicity, lidocaine cannot be empirically recommended for suspected aural insect foreign bodies.

Due to the presence of a live insect it would be difficult for EMS responders to examine for perforation of the tympanic membrane, even if an otoscope were available. In a study by Sousa et al that evaluated the clinical signs suggesting a perforated tympanic membrane, it was found that hypoacusis was the most frequent presenting symptom at 93.5% of the evaluated ears.^13^ Tinnitus was second at 16.4% of the sample population, followed by otorrhea at 9.4% and otalgia at 4.5%.^13^^,15^ If any of these symptoms are present, it would be recommended that lidocaine not be used, with prompt transport to the nearest ED the most appropriate course of action.

Once the insect has been killed, approaches to removal can vary depending on available equipment, position of the insect, and clinician preference. A strong light source and an otoscope are considered standard equipment for visualization. Alligator forceps, cerumen loops, Frazier suction tips, hooks, curettes, and tweezers have been suggested for foreign body removal, subject to availability.^8^,^10^ Referral to an otolaryngologist should be considered after a failed attempt at removal if the patient is immunocompromised or if there is pre-existing ear canal injury. Following removal, use of non-steroidal anti-inflammatory drugs for pain control and fluoroquinolone otic drops for infection prevention are recommended.^3^

## CONCLUSION

While case reports have documented in-hospital approaches to patients with intra-aural insects, prehospital literature is minimal, and little training exists for EMS personnel. Mineral oil and lidocaine can both be used to euthanize an intra-aural insect in consultation with a medical command physician. The benefits of prehospital intervention include pain control, relief of vertigo, and decreased risk of tympanic membrane rupture; these benefits are amplified for patients with extended transport times. While the risks and benefits of prehospital intervention are multifactorial, consideration should be given to the role that prehospital personnel can play in the treatment of intra-aural insect foreign bodies.

## Figures and Tables

**Image:**
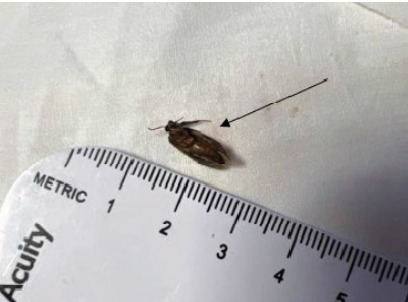
The foreign body insect removed from the patient’s auditory canal.
